# The impact of a rapid home test on telehealth decision-making for influenza: a clinical vignette study

**DOI:** 10.1186/s12875-022-01675-1

**Published:** 2022-04-13

**Authors:** Xinyan Cai, Mark H. Ebell, Rachel E. Geyer, Matthew Thompson, Nicole L. Gentile, Barry Lutz

**Affiliations:** 1 Department of Epidemiology and Biostatistics, College of Public Health, University of Georgia, Athens, GA 30601 USA; 2 Department of Family Medicine, University of Washington, Seattle, WA 98195 USA; 3 Department of Bioengineering, University of Washington, Seattle, WA 98195 USA

**Keywords:** Influenza, Decision-making, Decision thresholds, Home testing, Telehealth

## Abstract

**Background:**

Home testing for influenza has the potential to aid triage and management decisions for patients with influenza-like illness. As yet, little is known about the effect of the home influenza testing on clinical decision-making via telehealth. The goal of this study was to determine the clinicians’ decision thresholds for influenza and whether the availability of a home influenza test affects clinical decisions.

**Methods:**

We identified primary care physicians at 4 different sites in the US, largely via in-person continuing education meetings. Clinicians were asked for each vignette whether to treat empirically (“rule in”), ask the patient come to the clinic for further evaluation (“test”), or neither test nor treat (“rule out”). They were then given the results of a home influenza test, and were again asked to select from these three options. We measured the agreement of physician estimates of the likelihood of influenza with the probability based on a clinical prediction model. The test and treatment thresholds of influenza were determined based on mixed-effect logistic regressions.

**Results:**

In total, 202 clinicians made 570 sets of clinical decisions. Agreement between estimated and actual probability of influenza was fair. The test and treatment thresholds were 24% (95% CI: 22% to 25%) and 63% (95% CI: 58% to 65%) before revealing the actual likelihood of influenza. After providing the results of a home flu test the thresholds were similar, 26% (95% CI: 24% to 29%) and 59% (95% CI: 56% to 62%). However, approximately half of clinicians changed their cliical management decision after being given the home influenza test result, largely by categorizing more patients in the “rule out” and “rule in” groups, and reducing the need for in-person evaluation from 41% of patients to only 20%.

**Conclusion:**

In the context of a telehealth visit for a patient with influenza-like illness, we identified a test threshold of approximately 25% and a treatment threshold of approximately 60%. Adding the home influenza test results reduced uncertainty and significantly decreased the need for in-person visits.

**Supplementary Information:**

The online version contains supplementary material available at 10.1186/s12875-022-01675-1.

## How this fits in

Previous research has identified test and treatment thresholds of 5% and 5% for influenza management in the primary care setting, but it is not known whether these thresholds apply to the situation where a home test kit for influenza is available and the patient is being managed via telehealth. In that setting, we found that the test threshold was 25% and treatment threshold was 60%. We further found that adding information from a home influenza test to clinical signs and symptoms reduced the need for in-person evaluation by a physician by about 50%.

## Background

The burden of seasonal influenza is significant. According to the World Health Organization (WHO), seasonal influenza accounts for 3 to 5 million severe cases and causes 300,000 to 500,000 deaths every year around the world [1]. Each year in the United States alone there are between 140,000 and 810,000 influenza-related hospitalizations, and between 12,000 and 61,000 deaths [2].

Tests that allow users to collect their own specimens and conduct assays at home (which we will refer to as “home tests”) have been developed both for diagnosis and to monitor chronic diseases (e.g. diabetes mellitus) [3–5]. The home testing of self-collected respiratory specimens would be a novel approach to diagnosing and treating acute respiratory infections [6]. For example, rapid influenza diagnostic tests (RIDTs) could potentially be used by patients to diagnose influenza infection at home [7–10]. Indeed, self-collection of nasal swabs has been shown to be highly acceptable, feasible, and to have equivalent accuracy to staff-collected specimens among various populations [6, 11, 12]. RIDTs can help to minimize unnecessary diagnostic tests, reduce inappropriate antibiotic use, and facilitate prompt antiviral treatment, especially as the latter is most helpful within 24 h of symptom onset [13–15]. However, to the best of our knowledge the impact of the results of home testing for influenza on clinical decision-making has not been previously studied.

**Fig. 1:**
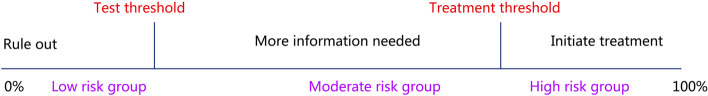
Illustration of the threshold model. Arrows indicate conceptual directions of “downward” and “upward” when a decision changes

The threshold model of decision-making initially proposed by Pauker and Kassirer suggests two decision thresholds: the test threshold and the treatment threshold (Fig. 1) [16]. Applied to influenza, if the probability that a patient has influenza is below the test threshold, then no testing or treatment is required; if the probability is above the treatment threshold, clinicians may consider empiric treatment; and if the probability falls between these two thresholds, more information is needed. Once decision thresholds are identified, clinical prediction rules could be designed to identify low-risk, moderate-risk, and high-risk groups of patients for influenza that reflect these thresholds [17].

In a previous study [18] one of the authors developed a novel approach for the estimation of decision thresholds by presenting physicians with a series of clinical vignettes that represented a realistic range of probabilities of influenza. For each vignette, physicians were asked to decide the probability of the disease and make a decision to rule out influenza, order a rapid test, or make the diagnosis and potentially initiate treatment. The “tipping point” for a decision to rule out influenza versus obtaining more information was the test threshold, while the tipping point for a decision to gather more information versus initiating treatment defined the treatment threshold. For the diagnosis of influenza in outpatient adults, the test threshold was estimated to be 5% and the treatment threshold 55% in the US primary care physician sample, with higher thresholds in a group of Swiss physicians. The current study uses this technique to explore physician decisions via telehealth regarding testing and treatment for influenza in the context of the availability of a home influenza test. The objective is to understand whether providing additional information in the form of a home rapid influenza test result affects physician decision-making and reduces the need for in-person evaluation.

## Method

### Participants

A convenience sample of primary care clinicians attending continuing medical education (CME) courses at the Cleveland Clinic (Cleveland, OH), Piedmont-Athens Regional Medical Center (Athens, GA), and Oak Street Health (Chicago, IL) was invited to participate in the study (these were all conducted in person prior to the COVID-19 pandemic). The vast majority of participants were not academicians and were in full-time clinical practice. We also used a convenience sample of primary care clinicians through the WWAMI (Washington, Wyoming, Alaska, Montana, Idaho) region Practice and Research Network (WPRN) and collected data via online questionnaires in the summer of 2020, during the COVID-19 pandemic. All surveys were completed anonymously and each IP address could only be used once for the online survey to prevent duplicate responses.

### Study design

After giving informed consent, each clinician was asked to provide demographic information, medical specialty and training (family medicine, internal medicine, physician assistant, or nurse practitioner), years in practice, practice site (primary care, urgent care, emergency department, or other sites), and the type of point-of-care flu test used in their practice (molecular or polymerase chain reaction [PCR] point of care test, older rapid antigen test, or none).

Each participant was then presented with three scenarios using a paper survey if in person or an online survey using REDCap if during the pandemic. Each scenario included a different combination of symptoms and disease prevalence, and each presented a patient with a different probability of influenza based on a published influenza risk score (the probability was not disclosed to the clinician) [19]. This risk score was based on 4 symptoms (acute onset, fever + cough, chills or sweats, and myalgias) and did not require physical examination, making it ideal for a telehealth simulation. The score classified patients into 3 risk groups with probabilities of influenza of 8% in the low risk group, 30% in the moderate risk group, and 59% in the high risk group. Clinicians were told that the context was a telehealth consultation with the patient, and were told that the overall prevalence of influenza among persons with influenza-like illness in the community was either 10% (shoulder season))or 30% (flu season). To avoid introducing bias or additional variation based on age, sex, or duration of symptoms, each scenario presented a 35-year-old woman who had the onset of symptoms 24 h ago. Each clinician was then asked to estimate the likelihood of influenza (0 to 100%) for each scenario and to select from one of three options:

- You feel that influenza is unlikely and recommend symptomatic treatment at home.
- You ask the patient to come to your office later today to be evaluated by you.
- You feel that influenza is likely enough to make the diagnosis and start treatment over the phone.

These options are consistent with being below the test threshold, being between the test and treatment thresholds, or being above the treatment threshold respectively in the threshold model. The initial estimate by the clinician of the probability that the patient had influenza, prior to being given the results of the home rapid test for flu, was labeled as the “pretest probability” of influenza. See Appendix Box 1 for an example of the clinical vignettes.

In the second stage, after clinicians completed their estimate of the pretest probability of influenza, they were asked to fold the paper survey back to reveal the actual likelihood of influenza based on the validated clinical prediction rule, which we refer to as the “model-based probability” [19]. This was done rather than using their initial probability estimate in order to standardize the second decision. In the online version, clinicians were not allowed to go back to a previous page after the true probability of disease was revealed in order to prevent them from making changes to their previous choices. The clinicians were also told that the patient had used a home test for influenza (sensitivity 70% and specificity 95% based on a systematic review[20], cost: $20) and were given an updated probability of influenza based on the model-based probability and a positive or negative result of the home test; this was labeled the “post-test probability”. After they had been given the post-test probability of influenza for each patient, clinicians were again asked to choose from the same three management options to see if the updated estimate of the likelihood of influenza incorporating the home test result affected their decision-making.

### Data preparation

Data were only included if the clinician provided complete data for that scenario. If a clinician provided an estimated probability of influenza as an interval, for example “10%—20%”, the midpoint of this range was used. Rarely, clinicians provided an estimated probability as “ < 10%” or “ > 90%”; in that case we used the midpoint between 0% or 100% and their estimate as their estimated probability. For example, “ < 20%” would be 10%, and “ > 70%” would be 85%.

### Analysis

The characteristics of each clinician were summarized descriptively. The pre-test probability estimated by clinicians and the model-based probability of influenza based on the prediction model were compared using a calibration plot. The percentage of clinician pre-test probability estimates that were under-, over-, or correctly estimated compared to the model-based probability were also plotted. A “correct” estimate was defined as one that was within plus or minus 25% of the model estimate, e.g. if the model estimated probability was 20%, then a clinician estimate between 15 and 25% would be considered correct.

The test and treatment thresholds were determined by adapting the method from a previous study [18]. Logistic regression analysis was used to estimate two pairs of test and treatment thresholds, one using the clinician’s initial management decision and pretest probability estimate (“prior thresholds”) and one for the updated management decision and post-test probability of influenza informed by the home test result (“posterior thresholds”). The confidence intervals of the test threshold and treatment threshold were calculated using a covariance matrix for the estimated coefficients in the model [21]. We also adjusted the prior and posterior threshold models by years of medical practice (< = 10 versus > 10), practice sites (primary care versus non-primary care), specialties (family medicine vs. non-family medicine), the type of point-of-care influenza test used in the practice (molecular PCR vs. older rapid antigen test), and the survey type (online versus in person) in order to compare subgroups.

The clinical management decisions before and after the provision of the post-test probability informed by home testing were compared in a reclassification table. We hypothesized that reclassification was more likely if the pre-test probability and the post-test probability fell on the opposite side of a decision threshold. Therefore, we also stratified reclassifications by this factor.

All statistical analyses were performed using R software, version 3.0.2 [22]. We applied the mixed-effect logistic regression by using the glmer() function from the lme4 package.

### Ethical approval

This study was approved by Human Subjects Committees of the University of Georgia and the University of Washington (STUDY00009003), and all participating clinicians provided written or electronic informed consent.

### Funding

This study was funded by Gates Ventures. The funder had no role in the design of the study; collection, analysis, and interpretation of data; and the decision to approve publication of the finished manuscript.

Gates Ventures

## Result

### Characteristics of participants

A total of 202 clinicians responded to the survey, of whom 190 completed the entire survey (94%). Among the respondents, 79 were from the Cleveland Clinic (39.1%), 73 from the WPRN (36.1%), and the rest from Oak Street Health (15.8%) or Piedmont Athens Regional Hospital (9%).

Overall, clinicians provided their probability estimates and clinical decisions for 570 clinical vignettes. The demographic characteristics of the clinicians are summarized in Table 1. The majority of clinicians were in a primary care setting (85.6%); 66% were family physicians and 52% had been in practice for more than 10 years. There was also a fairly even split between clinicians using molecular or PCR (38.1%) and those using the older rapid antigen test (36.6%) as the point-of-care test for influenza; 15.3% reported not using a rapid influenza test in their office. Table 2 summarizes the 12 clinical vignettes, the pre- and post-test probabilities, and the initial clinician decision for each one.

**Table 1:** Baseline characteristics of the participating clinicians

Characteristic (*n* = 202)	*n* (%)
**Training or specialty**	
Family Medicine	134 (66.3%)
Nurse Practitioner	28 (13.9%)
Internal Medicine	17 (8.4%)
Physician Assistant	5 (2.5%)
More than one specialty	3 (1.5%)
Other	8 (4%)
No response	7 (3.5%)
**Time in practice, years**	
< = 5	41 (20.3%)
6 to 10	40 (19.8%)
11 to 20	48 (23.8%)
> 20	57 (28.2%)
No response	16 (7.9%)
**Clinical setting**	
Primary care	173 (85.6%)
Urgent care	4 (2%)
Other	14 (7%)
No response	11 (5.4%)
**Point of care influenza test**	
Molecular or PCR	77 (38.1%)
Older rapid antigen test	74 (36.6%)
None	31 (15.3%)
No response	20 (10%)
**Practice Site**	
Cleveland Clinic	79 (39.1%)
Oak Street Health	32 (15.8%)
Piedmont Athens Regional	18 (9%)
University of Washington	73 (36.1%)
**Type of survey**	
Written	129 (63.9%)
Online	73 (36.1%)

**Table 2:** Description of the 12 clinical vignettes and their associated pretest and post-test probabilities, as well as the initial physician decisions

					**Initial Physician Decision**		
**Patient symptoms in vignette**	**Pretest probability of influenza**	**Community prevalence of influenza**	**Rapid flu test result**	**Post-test probability**	**Rule-out**	**Test**	**Treat**
Cough but no fever, chills/sweats or myalgias	3%	30%	Negative	1.0%	22	14	1
Cough but no fever, chills/sweats or myalgias	3%	10%	Positive	30%	22	1	0
Cough and chills/sweats, but no fever or myalgias	5%	10%	Negative	1.7%	22	6	0
Cough and chills/sweats, but no fever or myalgias	5%	10%	Positive	42%	29	8	0
Cough and chills/sweats, but no fever or myalgias	15%	30%	Negative	5.3%	21	16	0
Cough and chills/sweats, but no fever or myalgias	15%	30%	Positive	71%	12	11	0
Cough and myalgias, but no fever or chills/sweats	30%	10%	Negative	12%	15	20	2
Cough and myalgias, but no fever or chills/sweats	30%	30%	Positive	86%	17	7	4
Cough, fever, and chills/sweats but no myalgias	45%	30%	Negative	21%	1	21	6
Cough, fever, and chills/sweats but no myalgias	45%	30%	Positive	92%	1	25	11
Cough, fever, and myalgias but no chills/sweats	63%	30%	Negative	35%	1	11	11
Cough, fever, and myalgias but no chills/sweats	63%	30%	Positive	96%	1	18	18

### Estimation of test and treatment thresholds

The test and treatment thresholds *before* being shown the influenza home test result (prior probability) were 23.7% (95% CI: 21.9% to 24.7%) and 62.8% (95% CI: 58.3% to 65.3%), respectively (Table 3 and Fig. 3). The test and treatment thresholds based on clinician estimates of the likelihood of influenza *after* being given the results of the home influenza test (posterior probability) were 26.3% (95% CI: 23.8% to 29.1%) and 59.4% (95% CI: 56.3% to 62.4%) respectively. Thus, the decision thresholds were stable even with new information.

**Table 3:** Test and treatment thresholds, before and after being given results of the home test for influenza

	Before being given the home test result		After being given the home test result	
	Probability of influenza (95% CI)	*p*-value	Probability of influenza (95% CI)	*p*-value
***Test threshold***				
**Overall**	**23.7 (21.9 to 24.7)**		**26.3 (23.8 to 29.1)**	
**Practice type**		0.12		0.34
Primary care	24.3 (21.7, 26.5)		28.9 (26.2, 30.3)	
Non-primary care	16.3 (13.3, 24.3)		23.7 (21.6, 27.3)	
**Years in practice**		0.52		0.81
0–10	23 (20.3, 25.2)		27.7 (25.3, 30.2)	
> 10	25 (22.2, 27.3)		26.9 (23.7, 29.3)	
**Specialty**		0.57		0.24
Family physician	24.7 (21.8, 27.4)		29.8 (26.4, 32.3)	
Non-family physician	22.8 (20.2, 26.4)		25.6 (23.4, 30.2)	
**Diagnostic test**		0.22		0.54
Molecular or PCR	21.7 (18.4, 24.6)		25.2 (22.3, 28.4)	
Older antigen test	18.9 (16.2, 22.6)		27.3 (23.4, 30.1)	
**Survey type**		0.23	0.73	
Online	26.1 (23.6, 28.8)		27.0 (25.8, 29.2)	
Written	22.5 (20.2, 24.1)		26.0 (24.6, 27.4)	
***Treatment threshold***				
**Overall**	**62.8 (58.3 to 65.3)**		**59.4 (56.3 to 62.4)**	
**Practice type**		< 0.01		0.01
Primary care	63.5 (58.3, 67.6)		58.2 (52.3, 62.2)	
Non-primary care	53.9 (50.4, 56.4)		37.1 (33.3, 43.2)	
**Years in practice**		< 0.01		0.44
0–10	66.4 (62.5, 70.2)		60.6 (56.3, 65.4)	
> 10	60.9 (56.8, 64.1)		56.9 (52.7, 60.2)	
**Specialty**		0.83		0.1
Family Medicine physician	63.7 (60.4, 66.6)		57.8 (53.2, 61.6)	
Other clinician	63.5 (60.2, 65.8)		60.9 (56.2, 65.4)	
**Diagnostic test**		0.6		0.54
Molecular or PCR	62.2 (58.4, 64.2)		56.7 (52.2, 60.5)	
Older antigen test	63.7 (60.4, 65.4)		53.3 (50.8, 58.7)	
**Survey type**		0.12		0.46
Online	52.5 (50.3, 55.2)		54.0 (52.3, 56.1)	
Written	59.5 (54.6, 62.4)		59.5 (54.2, 52.6)	

For the stratified analysis of prior thresholds, the treatment threshold was significantly higher for primary care clinicians compared to non-primary care clinicians (63.5% vs. 53.9%, *p* < 0.01). The treatment threshold for clinicians practicing more than 10 years was lower than for those practicing less than or equal to 10 years (60.9% vs. 66.4%, *p* < 0.01). No significant differences between subgroups were found with respect to the test thresholds. For posterior thresholds, the treatment threshold was also significantly higher for primary care clinicians compared to non-primary care clinicians (58.2% vs. 37.1%, *p* = 0.01) (Table 3). The posterior test and treatment thresholds stratified by subgroups are presented in Appendix Table 1 and the stratified analyses are shown graphically in Appendix Figs. 1, 2 and 3.

**Fig. 2:**
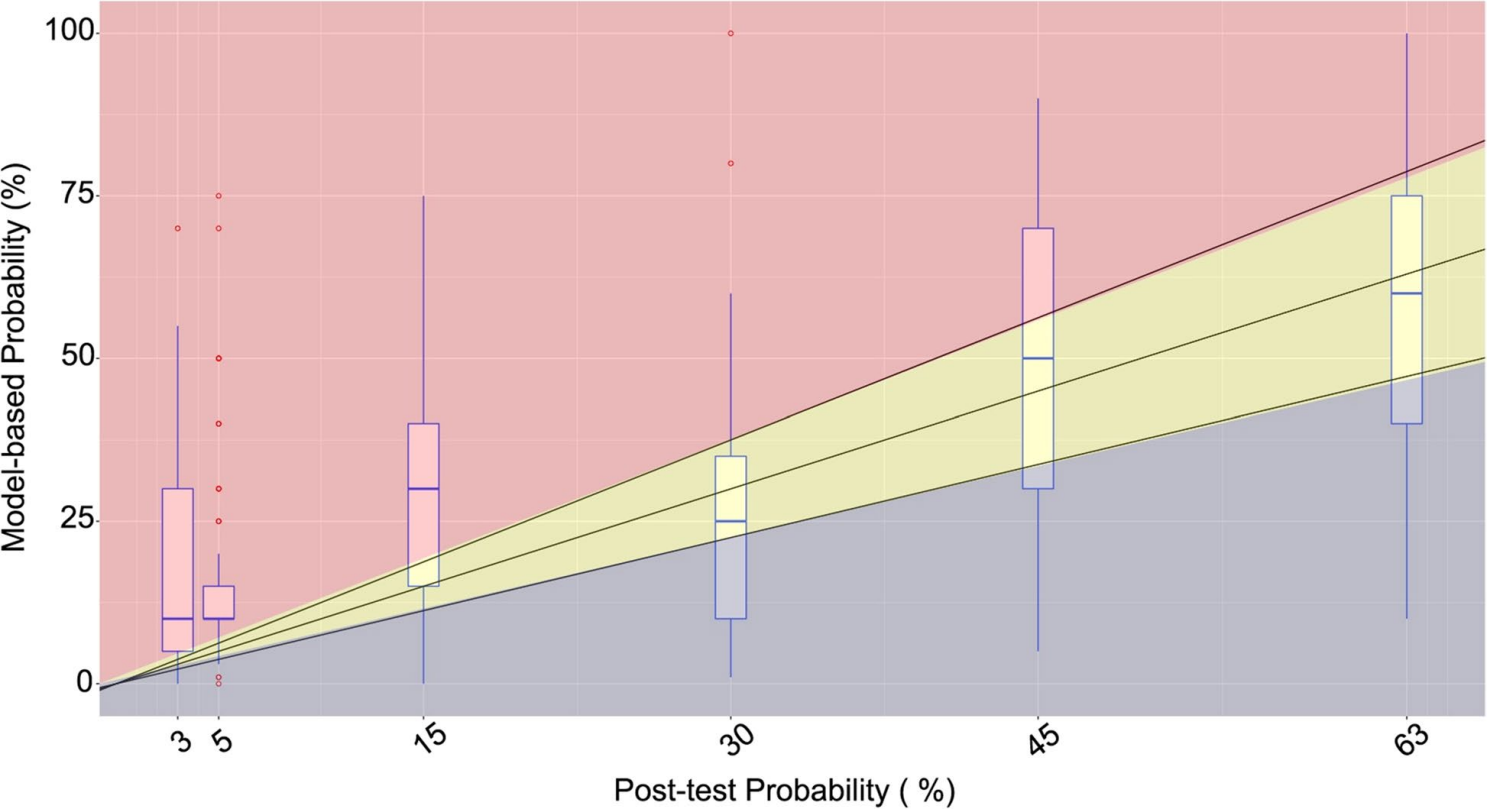
Clinician’s pre-test probability of influenza versus model-based probability of influenza estimated using the clinical prediction rule for each scenario. The boxplots represent the median, interquartile range, and the overall range of each clinical vignette. The middle black line indicates perfect agreement, and the yellow shaded region represents the area between 0.75 and 1.25 times of the model-based probability

**Fig. 3:**
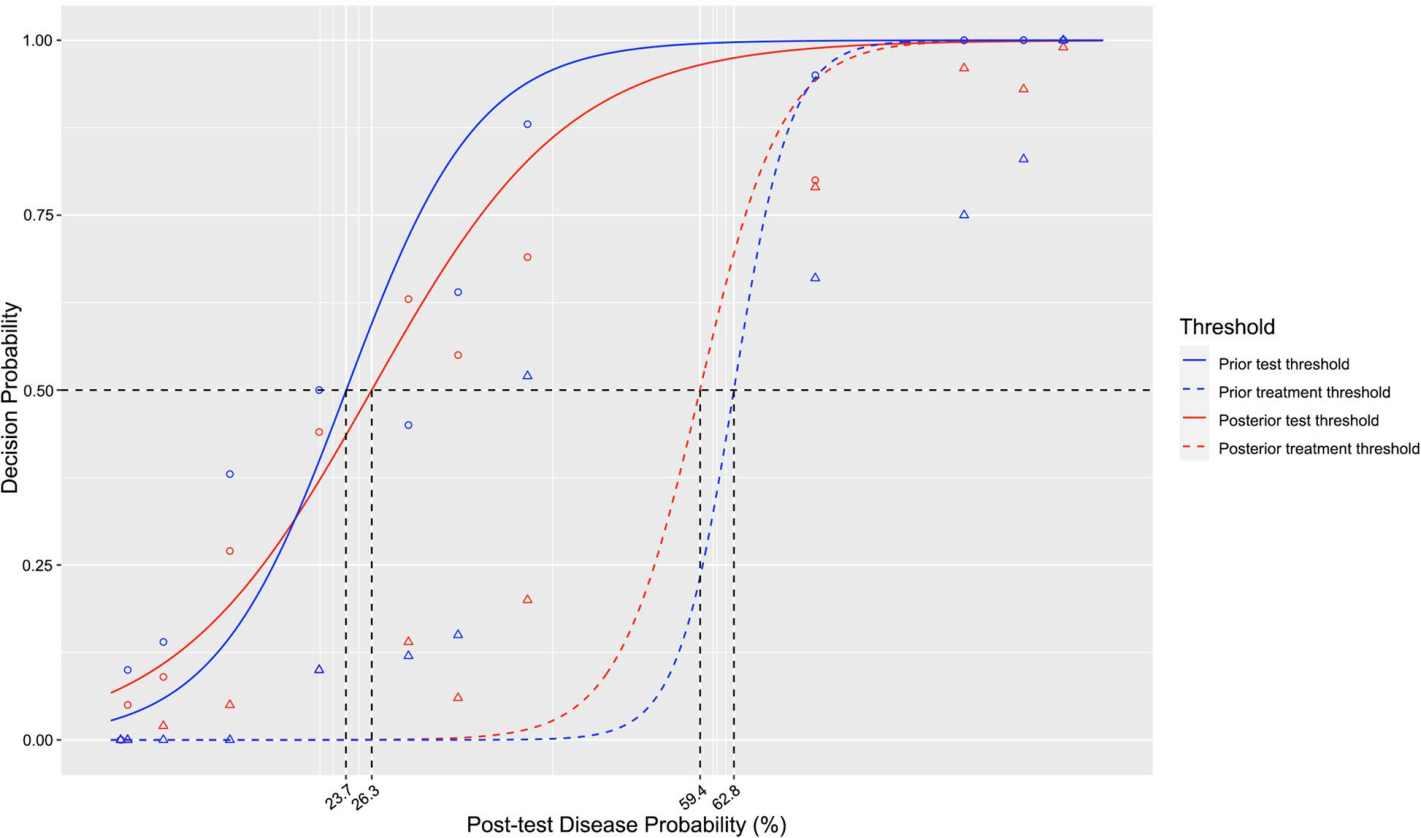
Prior test (blue solid line) and treatment (blue dashed line) thresholds based on pre-test probability, and posterior test (red solid line) and treatment (red dashed lines) thresholds based on post-test probability, obtained equaling to 0.5 the prior/posterior probabilities of not ruling out (test thresholds) and treating (treatment thresholds) estimated according to model 2. Points (circles and triangles) represent empirical frequencies of decisions according to the true/estimated disease probability

### Calibration between clinician estimate and model probability

The calibration between the initial pre-test probability estimated by clinicians and the model-based probability predicted by the clinical prediction rule is shown in Fig. 2. There was a moderate positive linear association between the two probabilities (r = 0.64). The majority of pre-test probability estimates by clinicians were higher than the model-based probability (56%) (red shaded area above the maximum range), while only 23% of the pre-test probability estimates fell within the range of ± 25% of the model-based probability interval (see yellow shaded area). Finally, only 21% of the pre-test probability estimates were lower than the model-based probability (blue shaded area below the minimum range); most of the underestimates were for higher disease probabilities.

### Impact of new information on clinician decision-making

Table 4 shows the reclassification of clinicians’ decisions before and after being given the probability of influenza based on the clinical prediction rule and the result of the home test for influenza. Just over half of clinicians changed their decision (295/570, 51.7%) after being given the test results. While the initial management decision was to have the patient come to the primary care office for further evaluation for 233 of 570 (40.9%) initially, after being given the post-test probability informed by the home test only 115 of 570 (20.2%) of clinicians still recommended the office visit, a reduction of 51%.

**Table 4:**
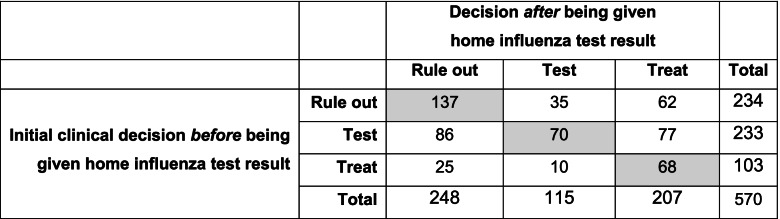
Reclassification table for decision-making before and after being given influenza home test results

Shaded numbers represent unchanged decisions

In terms of the direction of the post-test management decision, after the post-test probability informed by the home test was revealed, 121 of 570 decisions (21.2%) moved “downward” toward less testing and treatment, changing either from test to rule out (86/570, 15.1%), from treat to test (10/570, 1.8%), or from treat to rule out (25/570, 4.4%). A total of 174 of 570 of decisions (30.5%) moved”upward” toward more testing and treatment, specifically from rule out to treat (62/570, 10.9%), from rule out to test (35/570, 6.1%), or from test to treat (77/570, 13.5%).

We hypothesized that clinicians would be less likely to change their decision if the post-test probability was in the same region of the threshold diagram (Fig. 1) as the original pretest probability. Thus, in Table 5 reclassifications are stratified by whether or not the updated post-test probability remained in the same region (below the test threshold, between thresholds, or above the treatment threshold) or whether it moved to a different region. When the pre-test and post-test probabilities remained in the same region, only 57 of 185 decisions (31%) changed. However, when the post-test probability was in a different region than the pre-test probability, 238 of 385 decisions (62%) changed.

**Table 5:**
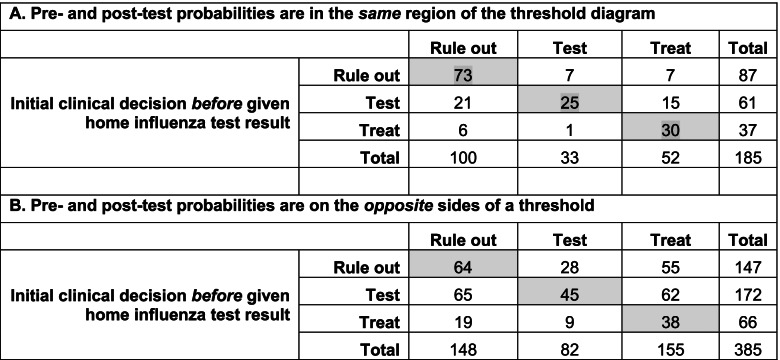
Reclassification table for decision-making before and after being given influenza home test results, stratified by whether the pre- and post-test probabilities were in the same or opposite regions of the threshold diagram ^a^

^a^ Regions are “Rule out” (below test threshold), “Test” (between thresholds) and “Treat” (above treatment threshold), see Fig. 1. Shaded numbers represent unchanged decisions

## Discussion

In the context of a telehealth visit for a patient with influenza-like illness, clinicians had a test threshold of approximately 25% and a treatment threshold of approximately 60%. These thresholds were stable after being given additional information in the form of the likelihood of influenza for the patient based on a clinical prediction rule and the results of a rapid home test for influenza. One would expect the clinical decision thresholds to be stable in the face of new information, so this supports the robustness of our approach to estimating decision thresholds. Importantly, while the thresholds were stable in the face of new information (the result of the home test for influenza) the clinical management decision often changed, with less than half as many patients now having an office visit recommended. This demonstrates that the results of home tests can inform clinical decision-making and potentially reduce healthcare visits and costs, while improving convenience for patients.

Clinicians’ initial estimates of the pretest probability of influenza infection differed markedly from probability estimates based on a previously published clinical prediction rule for influenza; only 23% of clinician estimates were within plus or minus 25% of the prediction rulel-based estimate. There was a tendency to overestimate the likelihood of influenza at lower probabilities with 56% of clinician estimates above the range of model-based estimates and only 21% below, perhaps due to a tendency to regress toward the mean. This shows that clinical decision support in the form of a clinical prediction rule plus a rapid influenza test has the potential to improve the accuracy of risk estimates and clinical decision-making.

Clinicians were asked to decide whether they were comfortable ruling out influenza (below the test threshold), would recommend further assessment or testing (between the test and treatment thresholds), or whether the diagnosis could be ruled in for possible empiric therapy (above the treatment threshold). Their initial decisions were 41% rule out, 41% test, and 18% treat, and reflected the pretest probabilities and community prevalence for each scenario. After being provided with the model-based estimate and home influenza test result, clinicians changed their clinical decision for 295 of 570 scenarios (52%), and were now 44% rule out, only 20% test, and 36% treat. This is consistent with the threshold model of decision-making: more information resulted in the ability to place more patients into rule out or treat groups, and reduced the need for in-person evaluation by 51%. An outpatient visit was initially recommended for 233 of 570 patients (40.9%), but after providing the model-based probability and result of a home influenza test, fewer than half that number (115 or 20.2%) would still have been asked to attend for in-person evaluation.

When reclassifications were stratified by whether the pretest and post-test probabilities are in the same or different decision regions of the threshold model (Fig. 1), as expected decisions changed much more often when the pretest and post-test probabilities were in different regions than when they were in the same region (62% vs 31%). This is consistent with what would be predicted by the threshold model and supports the validity of the test and treatment thresholds.

The treatment threshold was significantly higher for primary care clinicians compared to that for non-primary care clinicians (64% vs 54% for the pretest estimate). Instead of empirically prescribing any antivirals, even when the probability of influenza is estimated to be relatively high, primary care clinicians were more likely to recommend further evaluation for the patient. The treatment threshold was also higher if clinicians had been in practice for less than 10 years or were non-family physicians. This may reflect greater skepticism of the value of anti-influenza drugs among more recently trained clinicians. Another study  [23] found that the inappropriate prescribing of antibiotics was positively associated with time in practice. It has been found that late-career clinicians were more likely to prescribe longer courses of antibiotics compared to early-career clinicians [24]. In addition, the availability of diagnostic tools, as well as the degree of a clinician’s training, may also account for differences in the management options among clinicians.

### Comparison with existing literature

A previous study [18] identified a test threshold of 5% and a treatment threshold of 55% for patients presenting with influenza-like illness in the US. In that study, which used the same approach to threshold determination as our study, the vignettes placed the patient and physician in an office setting where a rapid test for influenza was readily available. Thus, the decision was whether to order a test. Our study found a higher test threshold, but a similar treatment threshold compared to the ones described in the previous study. This is likely because in our scenario of a telehealth visit, being above the test threshold and requiring more information would require having the patient make an appointment for an in-person visit, while in the previous study it just meant ordering a test in the clinical office.

### Strength and limitations

Strengths of the study include using realistic model-based scenarios to study clinician decision-making for patients with varying likelihoods for influenza. The sample of clinicians was from six different states, and was large enough to allow good precision for probability estimates. The study design allows us to simulate a realistic clinical encounter in the increasingly common telehealth context, investigate what happens when home testing for influenza becomes available, and help us understand its value and impact on decision-making.

However, this study has several limitations. Most importantly, this study used simulated patient cases and not real patients. However, care was taken to make the cases as realistic as possibly by creating them based on data from the largest outpatient study of patients with influenza-like illness to date. We would also argue that this was an important initial step to justify a larger clinical trial that could randomize patients to home test kit or no home test kit to determine the impact on patient outcomes. Such a trial could also explore whether patient characteristics such as age, sex, educational level, and disease severity influence use of a home test. Such a study could also compare the provision of explicit probabilities via an app or algorithm with the more widely used implicit probability of disease based on a physician’s overall clinical impression.

It is also possible that the post-test probability based on the model probability and home-based influenza test results may have systematically over- or underestimated the likelihood of influenza, which would bias clinicians’ decisions, and result in the estimated thresholds being higher or lower than they actually are. However, we found that thresholds were stable before and after providing new information, arguing against this being a problem.

The clinical vignettes for each scenario provided only limited information; for example, severity of symptoms was not reported. For example, a patient request for antivirals even with a negative test might influence the clinician’s decision. Moreover, even though patients reported symptoms for suspected influenza in the vignettes, clinicians might still rule out influenza based on their in-person assessement in clinical practice. In addition, only three management options were given for each vignette, which might not sufficiently capture all clinical management options, such as the use of a delayed prescription. However, given the timeliness needed for antiviral treatment, this is unlikely to have been an issue. Finally, some of the data having been collected during the pandemic may have biased clinician estimates away from influenza, although we saw no significant difference between thresholds in these two groups (Table 2).

## Conclusion

In the context of a telehealth visit for a patient with recent-onset influenza-like illness, we identified a test threshold of approximately 25% and a treatment threshold of approximately 60%. These thresholds were stable even when clinicians were given additional information about the probability of disease. Providing the results of a home-based influenza test resulted in about half of clinicians changing their management decision, and reducing the need for outpatient visits by 51%, which is potentially cost-saving for a health system and desirable for patients.

## Supplementary Information

**Additional file 1:** **Appendix Box 1. **Sample clinical vignette 

## Data Availability

Data are available from the authors upon reasonable request.

## Abbreviations

CI: Confidence interval
CME: Continuing medical education
COVID-19: Coronavirus disease 2019
IP: Internet protocol
PCR: Polymerase chain reaction
RIDT: Rapid influenza diagnostic test
US: United States
WHO: World Health Organization
WPRN: WWAMI (Washington, Wyoming, Alaska, Montana, Idaho) region Practice and Research: Network
