# Supplementary material for: The impact of a rapid home test on telehealth decision-making for influenza: a clinical vignette study
Source: BMC Prim Care. 2022 Apr 13;23:75. doi: 10.1186/s12875-022-01675-1 (PMC9006488; doi:10.1186/s12875-022-01675-1)

**Appendix**

**Appendix Box 1. Sample clinical vignette**

After answering questions about their training and practice site, participating physicians were given 3 clinical vignettes such as the one shown below. The scenarios were systematically varied to accurately describe patients with a range of influenza probabilities based on their signs and symptoms. Participants were then asked to make a recommendation for management:

**Scenario 1:** A 35-year old woman calls your office at 8:15 am. She reports a 24-hour history of cough and chills/sweats, but no fever or myalgias. She denies shortness of breath or hemoptysis and is otherwise healthy with no serious comorbidities. It is mid-flu season and overall about 30% of patients with acute RTI have influenza in your community.

Based on the prevalence, signs, and symptoms you estimate that her probability of influenza is _____%. Based on this probability, choose one of the following options:

A. You feel that the flu is unlikely and recommend symptomatic treatment at home

B. You ask her to come to your office later today to be evaluated by you

C. You feel that influenza is likely enough that you make the diagnosis and start treatment over the phone

They are then told the actual probability of influenza based on signs and symptoms using data from a large European study, and also the results of a home influenza test and the post-test probability of influenza based on the combination of signs, symptoms, and the test. They are then asked to make a decision based on this updated information:

A study found that patients with these signs/ symptoms have a 15% probability of flu. She adds that a home flu kit was negative, decreasing the probability to 5.3%. Based on this revised probability, you choose which of the following:

A. You feel that influenza is unlikely and recommend symptomatic treatment at home

B. You ask her to come to your office later today to be evaluated by you

C. You feel that influenza is likely enough that you make the diagnosis and start treatment over the phone

# **Appendix Table 1. Full model details for estimation of prior and posterior decision thresholds**

| **Thresholds estimation** | |
| --- | --- |
| **Prior test threshold** | |
| Intercept (a) | -3.5 |
| Pre-test probability (%) (b) | 15% |
| Prior test threshold (%) (-a/b) | 23.7% (95% CI 21.9% to 24.7%) |
|  | |
| **Posterior test threshold** | |
| Intercept (a) | -2.6 |
| Post-test probability (%) (b) | 10% |
| Posterior test threshold (%) (-a/b) | 26.3% (95% CI 23.8% to 29.1%) |
|  | |
| **Prior treatment threshold** | |
| Intercept (a) | -21.9 |
| Pre-test probability (%) (b) | 35% |
| Prior treatment threshold (%) (-a/b) | 62.8% (95% CI 58.3% to 65.3%) |
|  | |
| **Posterior treatment threshold** | |
| Intercept (a) | -14.3 |
| Post-test probability (%) (b) | 24% |
| Posterior treatment threshold (%) (-a/b) | 59.4% (95% CI 56.3% to 62.4%) |

**Posterior test and treatment thresholds according to post-test disease probability by subgroups**

Appendix Figure 1 Posterior thresholds for subgroups: ≤10 years in practice versus >10 years


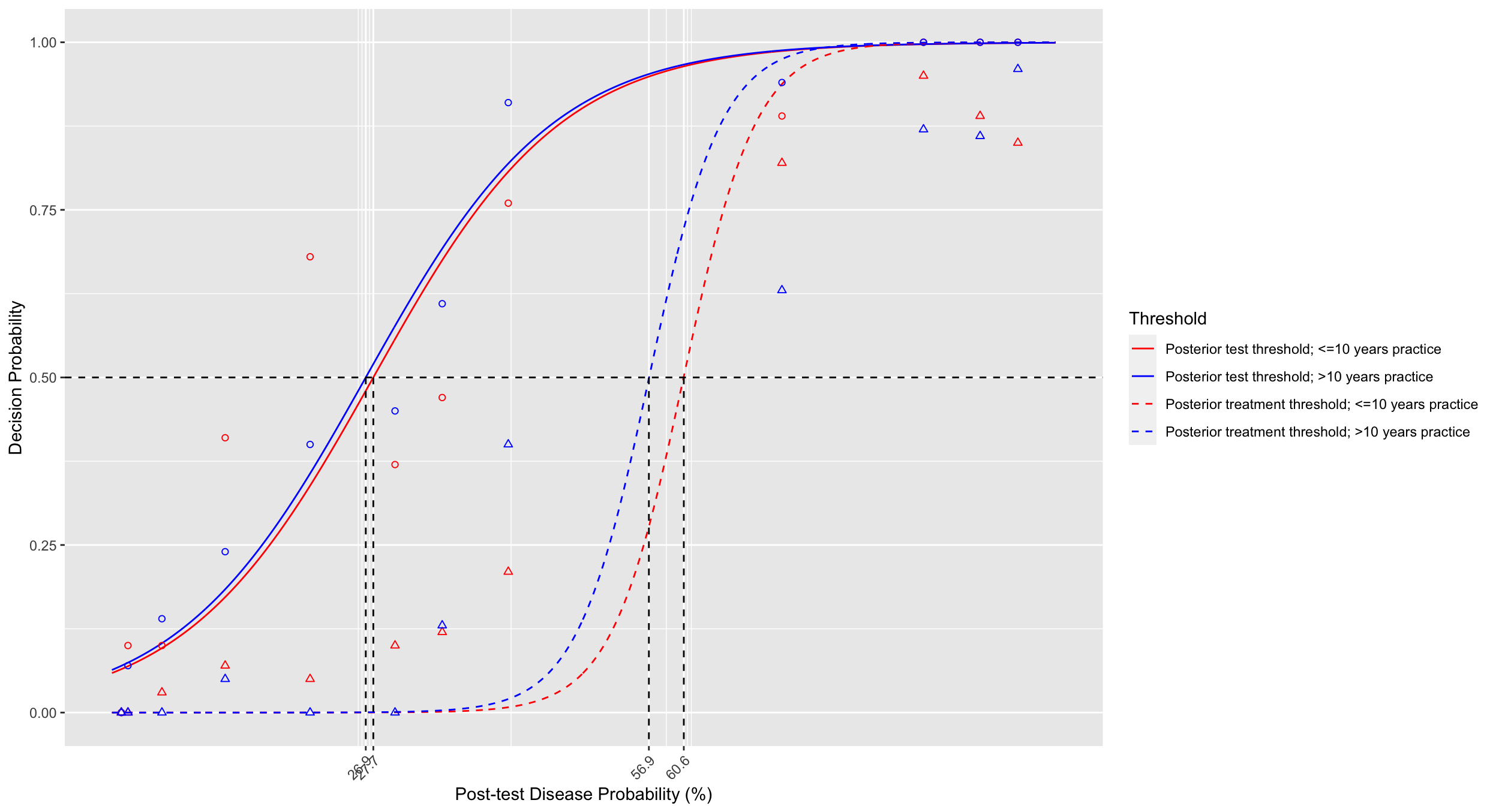


Appendix Figure 2 Posterior thresholds for subgroups: family physician versus non-family physician


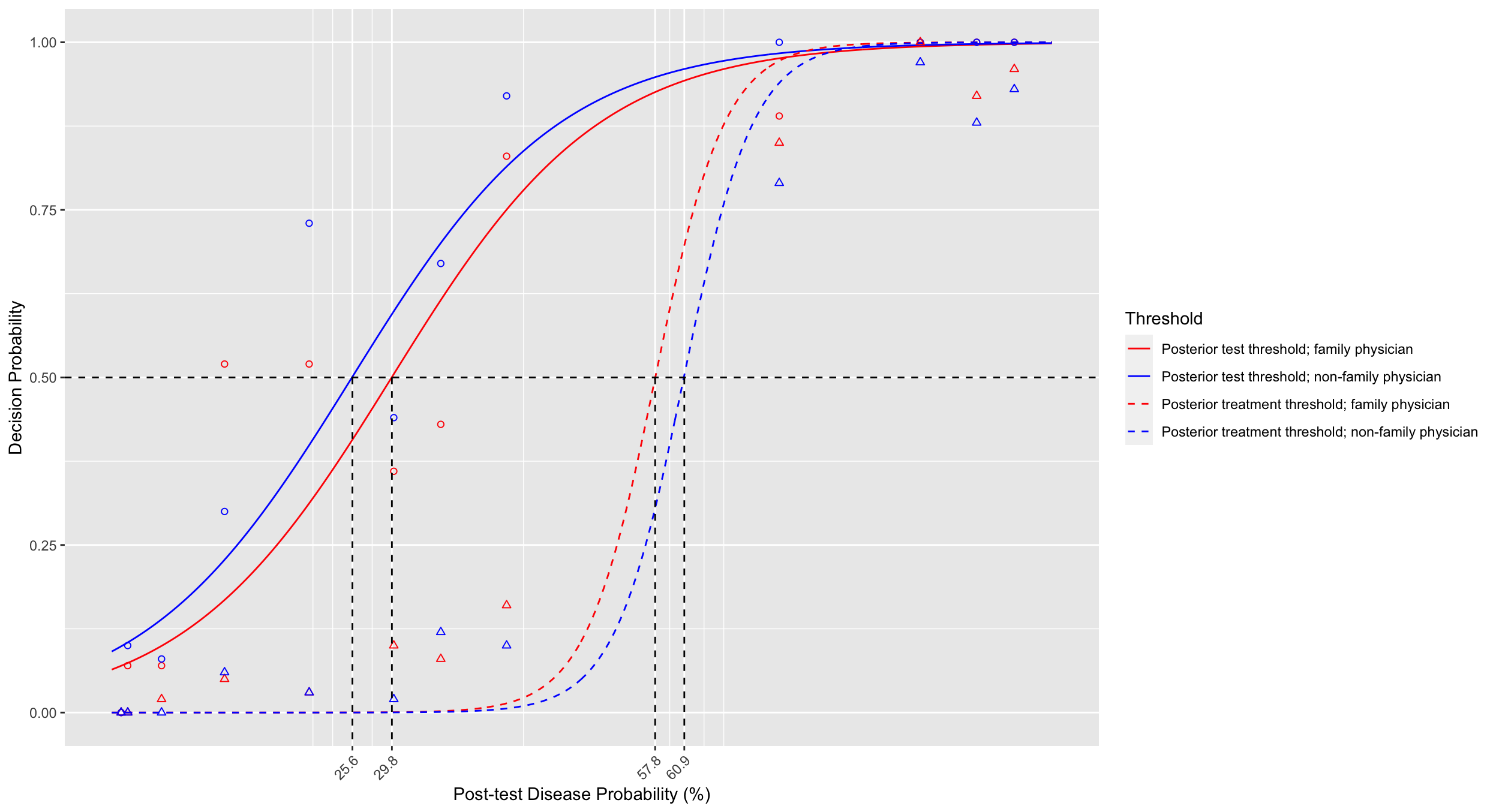


Appendix Figure 3 Posterior thresholds for subgroups: molecular or PCR versus older antigen test


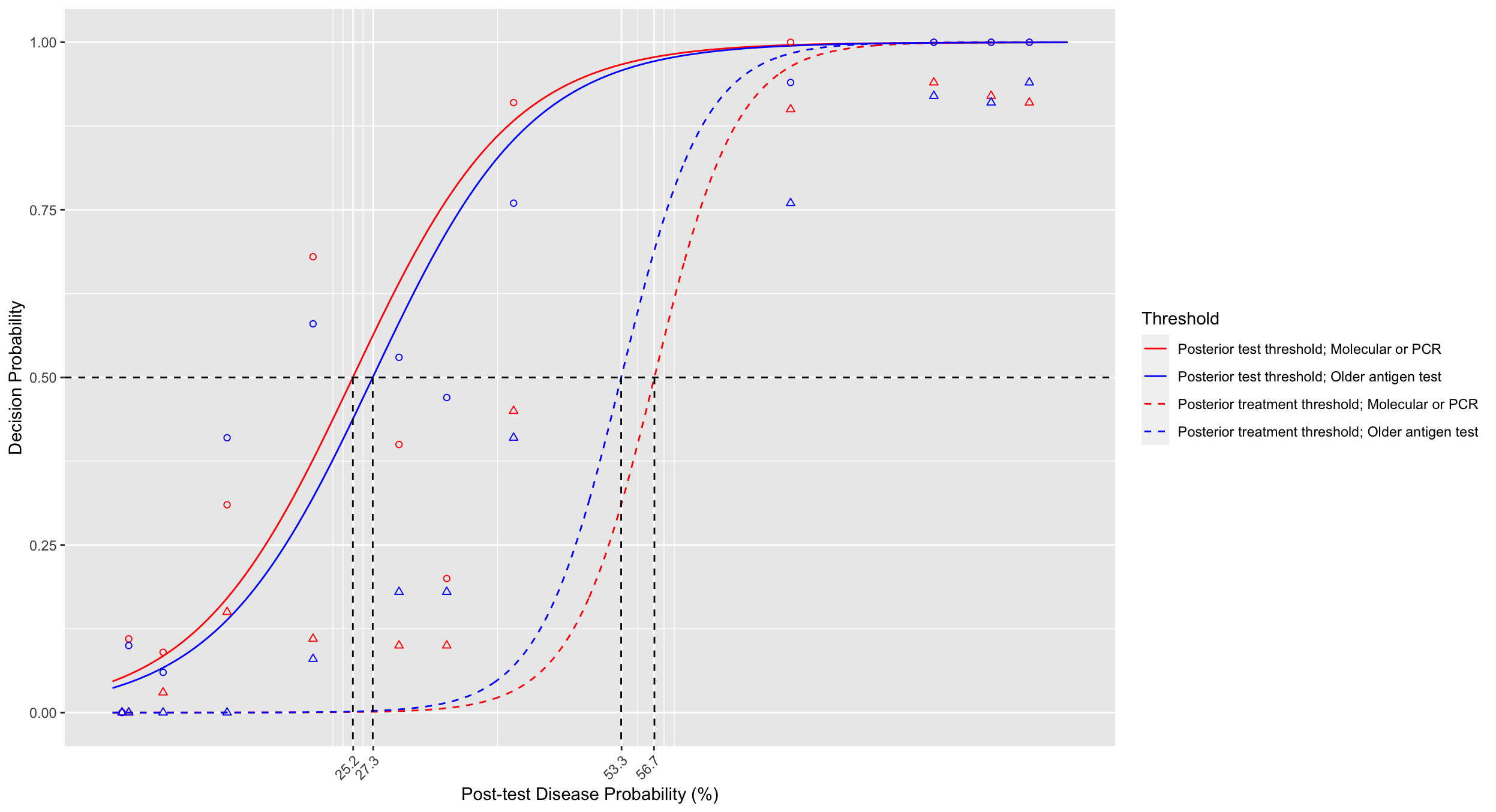


Figure 4 Posterior thresholds for subgroups: primary physician versus non-primary physician


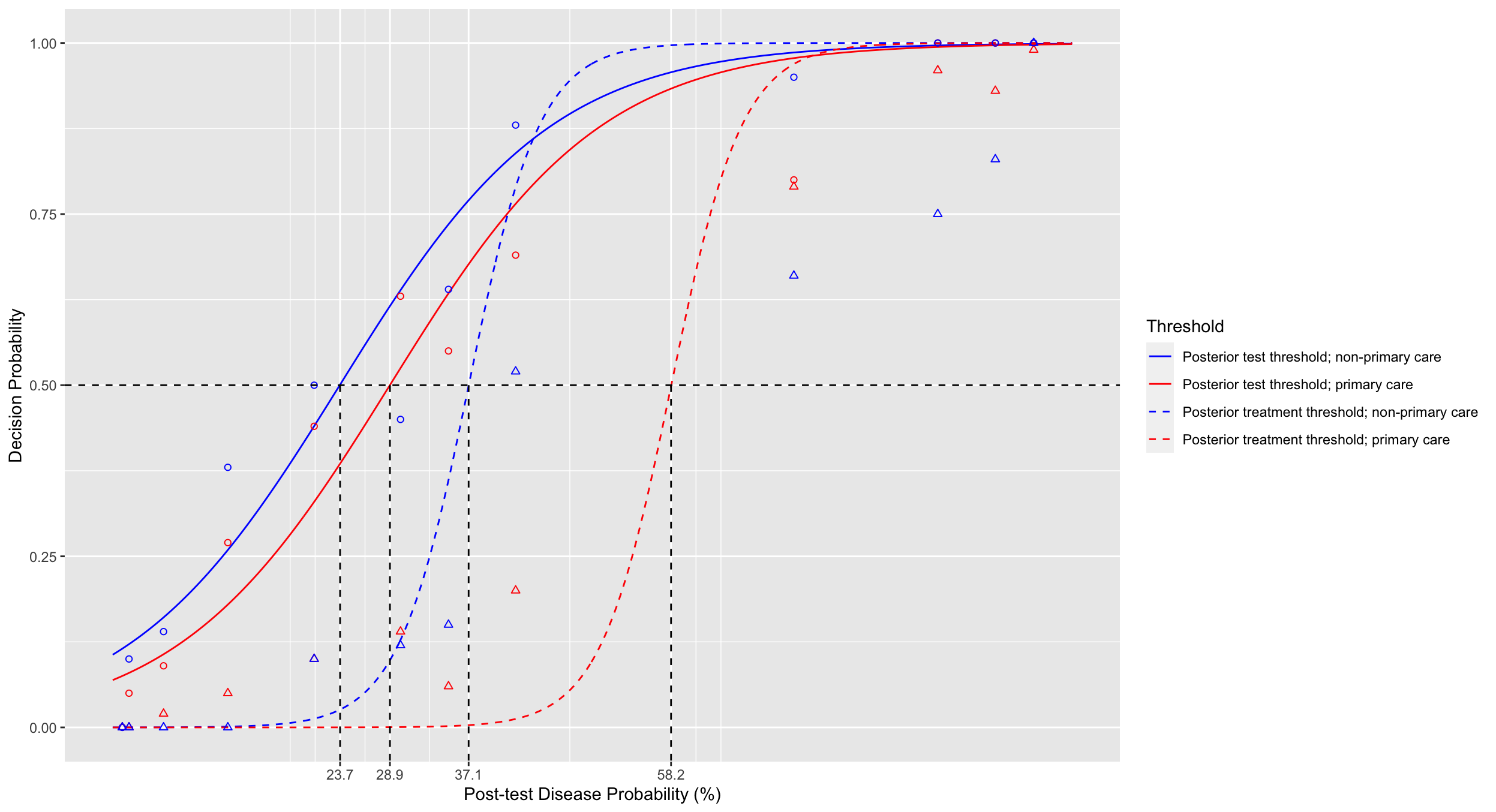

Supplement: Supplementary file 1 — Additional file 1: Appendix Box 1. Sample clinical vignette [file 12875_2022_1675_MOESM1_ESM.docx]
